# Behavioral Differences in the Upper and Lower Visual Hemifields in Shape and Motion Perception

**DOI:** 10.3389/fnbeh.2016.00128

**Published:** 2016-06-17

**Authors:** Giuseppe A. Zito, Dario Cazzoli, René M. Müri, Urs P. Mosimann, Tobias Nef

**Affiliations:** ^1^Gerontechnology and Rehabilitation Group, University of BernBern, Switzerland; ^2^ARTORG Center for Biomedical Engineering Research, University of BernBern, Switzerland; ^3^Division of Cognitive and Restorative Neurology, Department of Neurology, University Hospital Inselspital, University of BernBern, Switzerland; ^4^Privatklinik WyssMünchenbuchsee, Switzerland; ^5^University Hospital of Old Age Psychiatry and Psychotherapy, University of BernBern, Switzerland

**Keywords:** motion perception, perceptual asymmetries, shape perception, upper and lower hemifields, visual test

## Abstract

Perceptual accuracy is known to be influenced by stimuli location within the visual field. In particular, it seems to be enhanced in the lower visual hemifield (VH) for motion and space processing, and in the upper VH for object and face processing. The origins of such asymmetries are attributed to attentional biases across the visual field, and in the functional organization of the visual system. In this article, we tested content-dependent perceptual asymmetries in different regions of the visual field. Twenty-five healthy volunteers participated in this study. They performed three visual tests involving perception of shapes, orientation and motion, in the four quadrants of the visual field. The results of the visual tests showed that perceptual accuracy was better in the lower than in the upper visual field for motion perception, and better in the upper than in the lower visual field for shape perception. Orientation perception did not show any vertical bias. No difference was found when comparing right and left VHs. The functional organization of the visual system seems to indicate that the dorsal and the ventral visual streams, responsible for motion and shape perception, respectively, show a bias for the lower and upper VHs, respectively. Such a bias depends on the content of the visual information.

## Introduction

Human visual perception is not uniform in different regions of the visual field (Levine and McAnany, 2005). van Doorn et al. (1972) were among the first ones to study this topic in 1972. They found that the constitution of the receptor population in the retina strongly depends on the eccentricity, with higher density of photoreceptors in the center, and lower in the periphery. Perceptual differences go far beyond simple detection of light stimuli. Investigations of visual vertical asymmetries, for instance, historically favored the lower visual hemifield (VH) relative to the upper one. Talgar and Carrasco (2002) found, for example, higher perceptual accuracy when a behavioral task involving spatial resolution was performed in the lower VH. Similar results were found for contrast sensitivity (Carrasco et al., 2001) and motion processing (Levine and McAnany, 2005; Amenedo et al., 2007). However, an upper VH advantage was found in studies on visual search tasks (Previc and Naegele, 2001; Pflugshaupt et al., 2009) or detection of changes of letters in a word (Rutkowski et al., 2002). In the last 10 years, researchers extended the knowledge about perceptual asymmetries for low-level stimulus type (e.g., light sensitivity) to higher level visual processing (e.g., complex shapes, face processing). Studies using neuroimaging techniques and behavioral experiments showed that object-selective neurons are highly sensitive to stimulus position in the visual scene, and the ability to differentiate objects and faces based on their response is greatly variable across different positions in the visual field (Afraz et al., 2010; Kravitz et al., 2010). In line with these results, a recent experiment, conducted by Quek and Finkbeiner (2016), found that face processing is more accurately processed at above-fixation than below-fixation locations (Quek and Finkbeiner, 2016), and others found that face processing, in general, enjoys an upper VH advantage (Felisberti and McDermott, 2013).

In previous literature, two main explanations have been used in order to address such perceptual differences involving spatial attention and structural organization of the visual system. Drain and Reuter-Lorenz (1996), on the one hand, demonstrated, with a behavioral task involving length judgments of vertically oriented lines, that vertical biases are mediated by attentional factors, and are linked to the activation of the dorsal and ventral visual streams. On the other hand, Kravitz et al. (2013) showed, in studies on object perception, that, since the representation of the upper VH in the early visual cortex is contained below the calcarine sulcus (Sereno et al., 1995), presenting objects in the upper VH would enable more efficient transfer of information to the ventral stream in the temporal lobe. Transfer to the ventral visual stream from the dorsal stream, where information relative to the lower VH is initially projected, may take longer, and this is translated into vertical perceptual differences for object perception (Kravitz et al., 2010, 2013).

In the present study, we describe the results of novel behavioral tests, developed in order to target specific aspects of visual processing, and performed in different regions of the visual field. We used a previously developed visual test battery to selectively target perception of shape, orientation and visual motion (Zito et al., 2014), and implemented it in the four quadrants of the visual field. The novelty of our approach is that we used a real-time fixation control to account for eye movements, in the way that the tests could be performed only under central fixation. This aspect is crucial because, as suggested by Rezec and Dobkins (2004), when participants are free to move their eyes, the potential for a “scanning bias” (i.e., the tendency to begin a serial search in a particular region of space) is high, and this could mask location-dependent perceptual differences. Furthermore, the fixation control allowed to precisely place stimuli in specific regions of the visual field.

The following hypothesis was tested: perceptual asymmetries in the visual field are content-dependent, in the way that shape perception exhibits a bias in perceptual accuracy that favors the upper VH, while motion perception exhibits a bias in perceptual accuracy that favors the lower VH. No perceptual differences are expected when comparing right and left VHs.

## Materials and Methods

### Participants and Ethical Approval

Twenty-five healthy volunteers (6/14 right-handed men and 6/11 right-handed women, mean age = 28.0, *SD* = 4.6 years) were recruited to participate in the study. All subjects had a post-secondary education degree, normal or corrected-to-normal vision, normal alertness levels and were unaware of the hypotheses of the study.

The study was carried out in accordance with the latest version of the Declaration of Helsinki, and was approved by the Ethics Committee of the Canton of Bern, Switzerland.

### Experimental Design

Prior to the study, all subjects gave written informed consent. Subjects performed a practice session of the visual tests, followed by the actual testing session. Since few studies reported a relationship between visual asymmetries and alertness (Fimm et al., 2006; Heber et al., 2008), subjects also performed an alertness task (Zimmermann and Fimm, 2002), in order to control for the potential influence of this variable.

The duration of the experiment was about 1 h, comprising of 5 min for general assessment and training session, 5 min for the alertness task, and 4 min per each of the 12 subtasks of the actual session.

For the visual tests, subjects were seated on a height-adaptable chair at 60 cm from a 15.6″ screen (resolution of 1366 × 768 pixels) of an Intel^®^ Core^TM^ i5 (2.6 GHz) computer running Windows 7 Operating System (Microsoft Inc.,). Subjects placed their right index finger on the letter “L” button of a keyboard in front of them, and the left index finger on the letter “A” button. They were asked to perform a two-alternative yes/no forced-choice task, by pressing “L” for “yes” and “A” for “no”. The task was self-paced, but subjects were encouraged to react as fast as possible.

Three different subtasks, whose stimulus material was a modified version of the one described in previous studies (Zito et al., 2014, 2015a,b), were administered. Each subtask was repeated four times, in random order, each time in a different quadrant of the visual field. The level of difficulty of the single repetitions, in each subtask, was varied following a staircase paradigm with at least 20 steps (Wang et al., 2002; Pierce et al., 2013). The goal of the staircase paradigm was to measure the visual perceptual threshold (i.e., the minimum step of the staircase that could be correctly identified). For each subtask, the 20 steps were presented in random order but, in case of wrong judgment, the same step was presented four more times, again in random order, in order to make sure that the wrong answer was due to a perceptual limitation, and not due to other causes, such as distraction. Between two consecutive steps, a texture with random noise was shown for a duration of 1 s, in order to washout potential after-images effects across repetitions.

### Visual Tests

**Figure 1:**
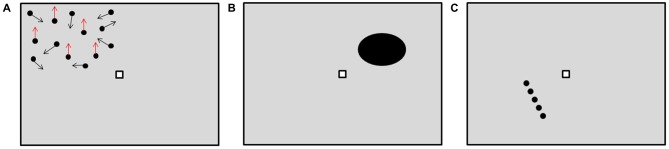
**Visual tests setup, with the three subtasks, as they appeared to the subjects. (A)** Movement Task performed, for example, in the top-left part of the visual field. For graphical reasons, only fewer dots than in the actual task are displayed. The arrows represent the direction of motion. In this case, the upward moving pattern is present, as shown by the red arrows. **(B)** Shape Task performed, for example, in the top-right part of the visual field. In this case, the presented shape is not a circle. **(C)** Orientation Task performed, for example, in the bottom-left part of the visual field. In this case the line is not vertically oriented.

In the first subtask, called Movement Task, a pattern of 200 small dots, with a diameter of 0.5° visual angles (VA) was presented at 4° VA eccentricity from a central fixation marker, placed at 0° VA (Figure 1A). A portion of the dots moved coherently upward, in order to convey a sense of coherent motion, while the remaining dots were given displacements from a flat distribution of directions spanning 360°, in order to create background noise (Rizzo and Nawrot, 1998). Subjects had to decide whether the pattern of dots moving coherently upward was present or not. For this subtask, the performance was measured as the inverse of the least dot coherence correctly detected by the subjects, defined as the minimum ratio between the number of dots moving coherently in a pattern and the total number of dots that was still recognized as a pattern. Smaller ratios resulted in a wrong judgment by the subjects. The performance was thus assessed according to the following formula:

$$Performance\;=\left(1-\frac{number\;of\;dots\;moving\;coherently\;upward}{total\;number\;of\;dots}\right)•100$$

For this subtask, the steps of the staircase were determined by the dots coherence, ranging from 0 to 60%, with a step size of 3%.

In the second subtask, called Shape Task, an ellipse with a major axis dimension of 3° VA was presented at 4° VA eccentricity from the fixation marker (Figure 1B). Here subjects had to decide whether the presented shape was a circle or not. The performance was assessed as the ratio between the major and the minor axes of the ellipse at perception threshold, normalized with respect to 100%. For this subtask, the steps of the staircase were determined by the ratio of the ellipse axes, ranging from 0.7 to 1, with a step size of 0.015. In the third subtask, called Orientation Task, a dotted line with length of 3° VA was presented at 4° VA eccentricity from the fixation marker (Figure 1C). Subjects had to decide whether the presented line was vertically oriented or not. The performance here was assessed as the actual angle of the line at perception threshold, normalized with respect to 100%. For this last subtask, the steps of the staircase were determined by the line angles, ranging from 85 to 95°, with a step size of 0.5°.

The visual tests were performed under central fixation, which was controlled by means of an eye-tracking system placed at the bottom of the computer screen (SMI REDm, SMI GmbH). If the subjects moved their gaze outside an allowed region of ± 2° VA from the central fixation marker, the visual stimuli disappeared, and they only reappeared when the central fixation marker was fixated again.

Tonic and phasic alertness were measured with the Test of Attentional Performance (TAP; Zimmermann and Fimm, 2002).

### Data Analysis

For the analysis of the results, the whole visual field was divided into VHs. The upper VH was compared to the lower one, and the right VH was compared to the left one. The performances in the upper and lower VHs, and in the right and left VHs, were computed as the mean value of the two performances from the respective quadrants. More in detail, the performances in the top-left and top-right quadrants, and in the bottom-left and bottom-right quadrants, respectively, were averaged in order to calculate the performance in the upper and lower VHs, respectively. Similarly, the performances in the top-left and bottom-left quadrants, and in the top-right and bottom-right quadrants, respectively, were averaged in order to calculate the performance in the left and right VHs, respectively.

Response latency was defined as the time to perform one subtask in one quadrant. Response latencies, in the upper and lower VHs, and in the right and left VHs, were computed in the exact same way as the performance.

The performances in the upper and lower VHs were entered in a repeated-measures analysis of variance (ANOVA) with within-subjects factors LOCATION (upper, lower) and SUBTASK (Movement, Shape, Orientation), and the between-subjects factor HANDEDNESS (right, left). Similarly, the performances in the left and right VHs were entered in a repeated-measures ANOVA with within-subjects factors LOCATION (left, right) and SUBTASK (Movement, Shape, Orientation), and the between-subjects factor HANDEDNESS (right, left). Tukey’s honestly significant difference (HSD) tests were used for *post hoc* comparisons. The same analyses were conducted for the response latency in the upper and lower VHs, and in the left and right VHs, respectively. The observed power was also studied.

Pearson’s correlations were calculated to study the relationship between the Movement and the Orientation Task, the Movement and the Shape Task, the Shape and the Orientation Task, respectively, over the whole visual field. Data were analyzed with STATISTICA 8.0 (StatSoft Inc.,). *Post hoc* power analysis was also conducted.

The anonymized dataset used for the analyses in this article is available on request. For further information on this database, please contact the corresponding author Tobias Nef (tobias.nef@artorg.unibe.ch) or Giuseppe Zito (giuseppe.zito@artorg.unibe.ch).

## Results

**Figure 2:**
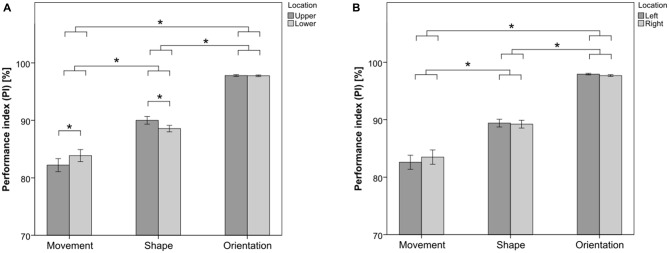
**Performance in the visual hemifields (VHs) for the Movement Task, the Shape Task and the Orientation Task, respectively. (A)** Mean performance in the upper and lower VHs. **(B)** Mean performance in the left and right VHs. Asterisks depict significant differences at *p* < 0.05, assessed with Tukey’s honestly significant difference (HSD) *post hoc* tests. Error bars represent the standard error of the mean.

The results of the three visual subtasks, when upper and lower VHs were considered, are shown in Figure 2A, while the results when left and right VHs were considered are shown in Figure 2B.

ANOVA on the performance in the upper and lower VHs showed a main effect of factor SUBTASK [*F*_(2,23)_ = 103.92, *p* < 0.01]. *Post hoc* analysis revealed that the Orientation Task reached the highest score among the three subtasks (*p* < 0.05 in both *post hoc* comparisons with the Shape Task and the Movement Task), with an accuracy over the whole visual field of 97.8% (± 0.6%), followed by the Shape Task (*p* < 0.05 in the *post hoc* comparison with the Movement Task), with an accuracy of 89.2% (± 2.7%), and finally by the Movement Task, with an accuracy of 83.1% (± 5.1%). No significant main effect of factor LOCATION was found [*F*_(1,24)_ = 0.06, *p* = 0.80]. A significant effect of interaction SUBTASK × LOCATION was also found [*F*_(2,23)_ = 8.48, *p* < 0.01]. *Post hoc* analysis revealed that the performance was better in the lower VH than in the upper VH for the Movement Task (*p* < 0.05). Conversely, the performance was better in the upper VH than in the lower VH for the Shape Task (*p* < 0.05). The performance for the Orientation Task did not differ between the upper and the lower VHs. No main effect of factor HANDEDNESS was found [*F*_(1,24)_ = 0.23, *p* = 0.64]. Neither effects of interaction HANDEDNESS × TASK [*F*_(2,23)_ = 0.10, *p* = 0.90], nor HANDEDNESS × LOCATION [*F*_(1,24)_ < 0.01, *p* = 0.95], nor HANDEDNESS × LOCATION × TASK [*F*_(2,23)_ = 0.36, *p* = 0.70] were found. *Post hoc* power analysis on the effect of interaction SUBTASK × LOCATION showed that, with a computed partial *η*^2^ of 0.27 and *α* = 0.05, the corresponding effect size *f* = 0.61. The *post hoc* power was finally 0.95, which can be considered adequate according to Cohen (1988).

In line with the results of the previous analysis, ANOVA on the performance in the left and right VHs showed a main effect of factor SUBTASK [*F*_(2,23)_ = 103.67, *p* < 0.01]. Neither significant main effect of factor LOCATION [*F*_(1,24)_ = 0.11, *p* = 0.74], nor significant effect of interaction SUBTASK × LOCATION [*F*_(2,23)_ = 1.12, *p* = 0.34] were found. No main effect of factor handedness was found [*F*_(1,24)_ = 0.21, *p* = 0.65]. Neither effects of interaction HANDEDNESS × TASK [*F*_(2,23)_ = 0.09, *p* = 0.92], nor HANDEDNESS × LOCATION [*F*_(1,24)_ = 0.04, *p* = 0.83], nor HANDEDNESS × LOCATION × TASK [*F*_(2,23)_ = 1.00, *p* = 0.37] were found.

ANOVA on the response latencies in the upper and lower VHs showed a main effect of factor SUBTASK [*F*_(2,23)_ = 44.72, *p* < 0.01]. Again, *post hoc* analysis revealed that the Movement Task, as compared with the other two subtasks, took the longest to be performed (*p* < 0.05 in both *post hoc* comparisons with the Shape Task and the Orientation Task), with a mean response latency of 2.53 s (± 1.08 s). No significant differences were found in the response latency between the Shape Task (1.33 ± 0.41 s) and the Orientation Task (1.11 ± 0.28 s). The main effect of factor LOCATION showed only a trend towards significance [*F*_(1,24)_ = 3.41, *p* = 0.08]. No significant effect of interaction SUBTASK × LOCATION was found [*F*_(2,23)_ = 0.85, *p* = 0.43]. No main effect of factor HANDEDNESS was found [*F*_(1,24)_ = 0.17, *p* = 0.68]. Neither effects of interaction HANDEDNESS × TASK [*F*_(2,23)_ = 0.02, *p* = 0.98], nor HANDEDNESS × LOCATION [*F*_(1,24)_ = 0.06, *p* = 0.80], nor HANDEDNESS × LOCATION × TASK [*F*_(2,23)_ = 0.95, *p* = 0.39] were found.

Similar results were found in the ANOVA on the response latencies in the left and right VHs, with a main effect of factor SUBTASK [*F*_(2,23)_ = 44.14, *p* < 0.01], no significant effect of factor LOCATION [*F*_(1,24)_ = 0.73, *p* = 0.40], and no effect of interaction SUBTASK × LOCATION [*F*_(2,23)_ = 2.12, *p* = 0.13]. No main effect of factor HANDEDNESS was found [*F*_(1,24)_ = 0.16, *p* = 0.69]. Neither effects of interaction HANDEDNESS × TASK [*F*_(2,23)_ = 0.02, *p* = 0.98], nor HANDEDNESS × LOCATION [*F*_(1,24)_ = 0.21, *p* = 0.65], nor HANDEDNESS × LOCATION × TASK [*F*_(2,23)_ = 0.42, *p* = 0.66] were found.

The correlation analysis showed that the Movement Task and the Shape Task positively correlated with the Orientation Task, (*ρ* = 0.38, *p* = 0.03 for Movement and Orientation, *ρ* = 0.52, *p* = 0.004 for Shape and Orientation), but not with each other (*ρ* = −0.04, *p* = 0.42).

## Discussion

The main finding of our study is that differences in perceptual accuracy across the visual field are content-dependent. In particular, motion perception was more accurate in the lower VH, while shape perception was more accurate in the upper VH. Such differences were not significant when the right and the left VHs were compared.

Focusing on motion perception alone, previous research found a lower VH advantage for space and motion processing (Levine and McAnany, 2005; Amenedo et al., 2007), which is line with our results of the Movement Task. Rezec and Dobkins (2004) for instance, found a lower VH advantage for motion segmentation, and interpreted it as an attentional bias in favor of the lower VH. They suggested that asymmetries in visual accuracy are attentional, rather than sensory, and are a consequence of uneven distribution of attention across the visual field (i.e., attentional weighting). However, others reported that the lower VH preference is not simply due to finer selective attention, but rather to visual constraints (Talgar and Carrasco, 2002; Levine and McAnany, 2005), and an attentional bias towards the lower VH *per se* would not explain the upper VH advantage found in our Shape Task.

An ecological theory that takes into account only the lower VH advantage observed in our Movement Task was found by Previc (1990). From an evolutionary point of view, the lower VH is more closely associated with the peripersonal space, where the hands interact with food, tools, and objects, and it requires fine analysis of the visual motion of the objects (Dessing et al., 2013). In contrast, the upper VH is more closely associated with the extrapersonal space, where stimuli are far away, and high accuracy of visual motion performance is not needed (Previc, 1990).

Concerning shape perception alone, studies on object recognition showed controversial results (Kravitz et al., 2008). In particular, these studies analyzed the size of the neuronal receptive fields (i.e., the range of retinal positions in which stimuli elicit responses), which is known to play an important role in position-dependence for object recognition, in the way that the larger the receptive field, the weaker the position dependence. They found that the receptive fields, in monkeys, increase in size along the ventral visual stream (Kobatake and Tanaka, 1994), spanning from 1° VA in V1 to more than 20° VA in the inferior temporal regions (Richmond et al., 1983). This suggested, thus, a position-independent recognition of objects in the later stages of visual perception. However, in another study, a higher heterogeneity of receptive field sizes in the inferior temporal cortex was found, ranging from 2.8° to 26° VA (Op De Beeck and Vogels, 2000), and thus suggesting the opposite, i.e., a position-dependent object recognition. Furthermore, receptive field size has been reported to vary with task demand (DiCarlo and Maunsell, 2003), and object representations are believed to arise from the responses of whole populations of neurons, rather than single neuronal cells (DiCarlo and Cox, 2007). This makes position-dependence of object recognition difficult to predict. Our behavioral results on the upper and lower VHs support the thesis of position-dependence for object recognition, because our Shape Task showed differential accuracy in subregions of the visual field.

An explanation that takes into account both the lower VH advantage for motion perception and the upper VH advantage for shape perception might be found in the functional organization of the visual cortex. Anatomical and physiological observations in monkeys indicated that, in early stages of visual processing, cells are separated in distinct pathways, and are selective for color, shape, movement, and orientation. Shape and color seem to be mainly derived from the parvocellular pathway, while space- and movement-selective components from the magnocellular pathway. The magno- and the parvo-cells, in turn, project into the dorsal and ventral visual streams, respectively (Livingstone and Hubel, 1988). Therefore, the dorsal stream, receiving predominantly magnocellular inputs, responds well to motion stimuli (Huberle et al., 2012; Pitzalis et al., 2013). Conversely, the ventral stream, with predominantly parvocellular inputs, is optimized for encoding information about color, shape and more in general, stationary stimuli (Merigan and Maunsell, 1993; Van den Stock et al., 2014). Moreover, many areas in the dorsal stream show a bias for the lower VH (Danckert and Goodale, 2001), and *vice versa*, areas in the ventral stream show a bias for the upper VH (Kravitz et al., 2010, 2013). In line with these aspects, our Shape Task, which is supposedly processed in the ventral stream, exhibited a bias in favor of the upper VH, while the Movement Task, supposedly processed in the dorsal stream, showed a lower VH advantage. The segregation between the mechanisms subtending the two subtasks was supported by the lack of correlation between the performances in the Movement and the Shape Task.

The Orientation Task did not show any vertical bias. Perception of visual orientation is known to occur in V1 (Roe and Ts’o, 2015). V1 receives input from the magno- and the parvo-cellular pathways, and projects into the two visual streams, as the correlation between Orientation Task and Shape Task, and Orientation Task and Movement Task, supported. However, recent studies on monkeys found no evidence of vertical asymmetries in V1 at this stage of visual perception, but only a decrease of visual accuracy from the foveal region to the periphery, suggesting, thus, that the neuronal populations in V1 follow a radial differentiation in processing accuracy, and the content-dependent vertical bias only occurs at later stages of visual perception (Palmer et al., 2012; Chaplin et al., 2013).

Perceptual differences in different regions of the visual field were also described in a comparison between the right and the left VHs. Studies in patients with unilateral lesions of the inferior temporal cortex (Biederman et al., 1997) and in macaques (Merigan and Saunders, 2004) suggest that there are two independent groups of neurons, each responsive to stimuli in only one VH. Therefore, visual accuracy, in the right and left VHs, should show a certain degree of position dependence. Our results did not confirm this pattern, because no differences were found between the right and left VHs, in any of the proposed subtasks. Nevertheless, it is possible that, in healthy subjects, this horizontal bias is not as pronounced as the vertical one, and our measurement method was not able to detect such small differences.

A final remark has to be made concerning the differences in the performance in the three subtasks over the whole visual field. The Orientation Task reached the highest accuracy, followed by the Shape Task, and finally by the Movement Task. This suggests that the difficulty level was not identical across the three subtasks, but the Orientation Task was the easiest to perform, the Shape Task was the second easiest, and the Movement Task the most difficult. Previous research showed that motion and shape are complementary features of visual objects, and both contribute to achieve efficient object recognition (Schultz et al., 2008), but motion was found to play a minor role in object discrimination compared to shape (Vuong et al., 2012). This might suggest that shape perception is somewhat more accurate than motion perception and in line with our results, a task involving pure motion discrimination, like our Movement Task, showed lower accuracy than a task with pure shape perception, like our Shape Task. The results of the response latency seem to indicate a similar pattern, because the time to complete the Movement Task was significantly higher than the time to complete the other two subtasks. However, the time spent on the Movement Task cannot be only attributed to the difficulty of the task itself, but also to its nature, as motion detection involves by definition a temporal component (Sekuler et al., 1975) and thus, a certain processing delay which is not present in the detection of static objects.

In conclusion, the main strength of the present study is that, to the best of our knowledge, for the first time a model to test behavioral differences in motion, shape, and orientation perception within the same paradigm was developed. From the technical point of view, the fixation control used in our experiments resulted to be advantageous, because perceptual differences due to a “scanning bias” could be excluded, and the visual stimuli could be exactly placed in precise regions of the visual field.

A possible limitation of our approach is that the visual tests were performed on a 15.6″ computer screen and especially for the Orientation Task, the borders of the screen might have been used as a reference for vertical lines. However, given the distance between the participants’ eye and the screen, and the central fixation maintained for the entire duration of the experiment, the upper and lower borders of the screen were located at about 9.5° VA, the right and left ones at about 16° VA. At such eccentricities, visual acuity is less than 30% of its maximum in the fovea (Hunziker, 2006), and an high impact on perception of visual stimuli can thus be excluded.

Another possible limitation relies on the different difficulty levels found for the three subtasks. It might be possible that, with equally difficult tasks, the effects shown in the present study would become more pronounced. As shown by Bankó et al. (2011), perceptual decision-making involves a task difficulty component and in turn, task difficulty can be manipulated by adding noise to the stimuli. Accordingly, a possible outlook would thus be the development of a novel paradigm, in which the difficulty level is systematically manipulated, for instance by embedding the stimuli in a texture of random noise, and adjusted, prior to the experiment, to the subjects’ individual performance.

## Author Contributions

GAZ designed the experiment, carried out the measurement, analysis and interpretation of the results. DC contributed to the discussion of the results. RMM and UPM gave substantial help in the conceptual development of the study. TN coordinated the study and helped with the experiment design. All authors contributed in reading, correcting and approving the final manuscript.

## Funding

This study was funded by the ARTORG Center for Biomedical Engineering Research (University of Bern, Bern, Switzerland), the Department of Neurology (Inselspital, Bern, Switzerland) and the University Hospital of Old Age Psychiatry and Psychotherapy (University of Bern, Bern, Switzerland).

## Conflict of Interest Statement

The authors declare that the research was conducted in the absence of any commercial or financial relationships that could be construed as a potential conflict of interest.
